# Determinants of Mortality from Cardiovascular Disease in the Slums of Nairobi, Kenya

**DOI:** 10.5334/gh.787

**Published:** 2020-04-10

**Authors:** Frederick M. Wekesah, Kerstin Klipstein-Grobusch, Diederick E. Grobbee, Damazo Kadengye, Gershim Asiki, Catherine K. Kyobutungi

**Affiliations:** 1 African Population and Health Research Center, 2^nd^ Floor APHRC Campus, Manga Close, Off Kirawa Road, Kitisuru, Nairobi, KE; 2 Julius Center for Health Sciences and Primary Care, University Medical Center Utrecht, Utrecht University, NL; 3 Department of Global Health and Population, Harvard T.H. Chan School of Public Health, Harvard University, Boston, MA, US; 4 Division of Epidemiology and Biostatistics, School of Public Health, Faculty of Health Sciences, University of the Witwatersrand, Johannesburg, ZA

**Keywords:** Cardiovascular risk, mortality, social determinants of health, education, employment

## Abstract

**Background:**

Cardiovascular diseases (CVD) comprise eighty percent of non-communicable disease (NCD) burden in low- and middle-income countries and are increasingly impacting the poor inequitably. Traditional and socioeconomic factors were analyzed for their association with CVD mortality over 10 years of baseline assessment in an urban slum of Nairobi, Kenya.

**Methods and results:**

A 2008 survey on CVD risk factors was linked to cause of death data collected between 2008 and 2018. Cox proportional hazards on relative risk of dying from CVD over a 10-year period following the assessment of cardiovascular disease risk factors were computed. Population attributable fraction (PAF) of incident CVD death was estimated for key risk factors. In total, 4,290 individuals, 44.0% female, mean age 48.4 years in 2008 were included in the analysis. Diabetes and hypertension were 7.8% and 24.9% respectively in 2008. Of 385 deaths recorded between 2008 and 2018, 101 (26%) were caused by CVD. Age (hazard ratio (HR) 1.11; 95% confidence interval (CI) 1.03–1.20, p = 0.005) and hypertension (HR 2.19, 95% CI 1.44–3.33, p <0.001) were positively associated with CVD mortality. Primary school education and higher (HR 0.57, 95% CI 0.33–0.99, p = 0.044) and formal employment (HR 0.22, 95% CI 0.06–0.75, p = 0.015) were negatively associated with CVD mortality. Controlling hypertension would avert 27% (95% CI 9%–42%, p = 0.004) CVD deaths, while if every member of the community attained primary school education and unemployment was eradicated, 39% (95% CI 5% – 60%, p = 0.026), and 17% (95% CI 5%–27%, p = 0.030) of CVD deaths, would be averted respectively.

**Conclusions:**

A holistic approach in addressing socioeconomic factors in the broader context of social determinants of health at the policy, population and individual level will enhance prevention and treatment-adherence for CVD in underserved settings.

## Introduction

Cardiovascular diseases (CVD) which comprise coronary heart disease (CHD), cerebrovascular disease (e.g. stroke), and ischemic heart attacks occurring in low- and middle-income countries (LMICs) are estimated at 80% of the global burden [1,2]. Sub-Saharan Africa (SSA) accounted for 5.5% of global CVD deaths (nearly one million deaths) in 2013 [3], a number that is expected to increase in the coming decade due to ageing, population growth, and epidemiologic transition currently being experienced in the region [3,4].

The SSA region is recording rapid urbanization, which has been linked to the increasing CVD burden [5,6]. The UN-Habitat estimates that about half of the population in SSA currently lives in urban areas, the number projected to double by the year 2050 [7]. Urban dwelling (living in cities and town areas far removed from rural areas) is divided into formal settlements (made up legally recognized and planned living arrangements) and informal settlements (made up of unplanned, mostly illegal and unrecognized shanties and temporary structures whose owners are without security of tenure). A majority of the urban dwellers live in informal settlements in slum-like environments which are characterized by poverty, lack of basic water and sanitation infrastructure and rampant crime [8]. Compared to the general population, slum residents are poor and are consequently disproportionately exposed to poor health and disease, and to enhanced risk for CVD [9,10]. Urban environments are known to predispose populations to CVD risk factors because these environments promote unhealthy diets [11], sedentary lifestyles, increased smoking and harmful consumption of alcohol [5,12].

Residents of slums also suffer from CVD risk factors that include high blood pressure, obesity, high blood cholesterol and diabetes [13]. Emerging evidence from LMICs indicate that socioeconomic factors that include education, wealth status and employment are correlated with CVD risk [14]. Socioeconomic status (SES) can influence behavioral risk factors, which in turn influence physiological risk factors that increase the risk for developing CVD [6,15]. Other studies have also shown that psychosocial stress, anger, anxiety and depression are associated with CVD in some settings in SSA [16]. In sum, different risk factors and exposures work together in an intricate fashion to cause CVD in individuals [17].

Data from previous research in low-resourced settings in Nairobi show a relatively higher prevalence of CVD risk factors in these settings when compared to the overall urban and rural populations in Kenya. In 2010, raised blood glucose/diabetes and raised blood pressure/hypertension among individuals aged 18 years and older was estimated at 5.3% and 22.8% respectively, with diabetes doubling to 10.5% in the 45- 54 age group [18,19]. In studies conducted between 2008 and 2012 in the same settings, central obesity, hypercholesterolemia, and hypertriglyceridemia was 12.3%, 10.3%, and 17.3%, respectively [20], while unhealthy diet, insufficient physical activity, harmful alcohol consumption and tobacco use was 57.2%, 14.4%, 10.1%, and 12.4%, respectively [21,22]. The accompanying levels of awareness, treatment and control of diabetes and hypertension was low [22]. Perception towards CVD risk and risk factors was also varied; this despite the fact that perception is known to influence the way individuals seek for, and adhere to treatment in such settings, and in other communities in SSA [23,24,25].

A SES gradient exists for CVD, with the poor being the worst affected [26]. However, little is known about the contribution of specific socioeconomic factors like education and employment to CVD mortality in the urban poor settings. These individual-level socioeconomic factors may reveal differences in the disease burden among urban slum dwellers, as has been documented in emerging evidence from LMICs [27] and in developed countries [28].

The current study investigated the contribution of physiological, behavioral and socioeconomic risk factors to CVD mortality in an underserved urban slum community in Nairobi, over a 10-year period (2008–2018) using data from a demographic surveillance system and from verbal autopsy cause of death. Evidence from this study may help in the design of strategies and programs for CVD prevention and control at the policy, population and individual levels by identifying and addressing key determinants of CVD mortality.

## Methods

### Data source and study setting

Data was obtained from the Nairobi Urban Health and Demographic Surveillance System (NUHDSS), operated by the African Population and Health Research Center (APHRC) since 2002. About 82,000 people resident in 33,000 households were part of the NUHDSS at the end of 2018. More on the NUHDSS is published elsewhere [29].

For each resident in the NUHDSS, a record is kept and updated three times annually during household visits (the frequency of household visits has been reduced to twice since 2015) on demographic events related to births, out-migration and in-migration, and death. Other household data include living arrangements and property ownership. Data on ‘residency’ of individuals in their community provides us the unique opportunity to conduct event-history analysis for each individual for the period they spend in the NUHDSS. Death, as an event, is also recorded if the individual dies in the NUHDSS, and the case is confirmed.

### The cardiovascular diseases risk factor study (2008)

In 2008, a cross-sectional study was conducted in the NUHDSS to assess the linkages between socioeconomic and socio-cultural factors, and perceived personal risk for CVD and health behavior in a slum setting (CVD 2008 study). The cross-sectional study provided baseline data for the current analysis. The CVD study adopted a stratified random sampling based on the World Health organization (WHO) STEPwise protocol [30] targeting 250 respondents in each of the following strata: sex (men and women), age-group (18–29, 30–39, 40–49, 50–59, 60 years and over), and slum of residence (Korogocho and Viwandani). A total of 5,470 individuals (3,018 men and 2,452 women) aged 18 years and older participated in the study.

## Measurements

### Self-reported measurements

Tobacco use and smoking, physical activity and disease history were self-reported. Individuals that reported engaging in 150 minutes/week moderate- and 75 minutes/week vigorous-intensive activities were considered physically active. Sedentary behavior was defined as any waking behavior characterized by sitting or reclining for more than seven hours each day, even when one is involved in moderate-vigorous physical activities.

### Physical measurements

Height was measured in centimeters using SECA™ height boards while the individual stood in an upright position on a flat surface. Body weight was measured in kilograms using calibrated SECA™ digital weighing scales.

### High blood pressure and hypertension

Blood pressure (BP) was measured using automated digital blood pressure devices (OMRON™). Using appropriate cuff sizes, three readings were taken on the left arm with the individual in a seated position, at one minute intervals. The average of the second and third readings was used for the current analysis. Raised blood pressure was defined as systolic blood pressure (SBP) ≥ 140 mmHg and/or diastolic blood pressure (DBP) ≥ 90mmHg. Hypertension was self-reported, based on a previous diagnosis by a physician, and/or if the individual was previously or currently on medication.

### Raised blood glucose, diabetes and hypercholesterolemia

A drop of blood from a finger prick was used to test for glucose and total cholesterol levels using the combined ACCU-CHEK™ Glucose, Cholesterol and Triglycerides (GCT) digital meter. In addition, fasting blood glucose and oral glucose tolerance tests were conducted. Raised blood glucose was based on WHO criteria for random capillary blood glucose ≥11.1 mmol/L, oral glucose tolerance test 2-hour post-load of ≥11.1 mmol/L, or fasting blood glucose of 7.0 mmol/L [31]. Total cholesterol levels were classified as ideal or high based on a cut-off of 5.2 mmol/L [32]. Diabetes was based on self-report of a previous diagnosis by a physician, and/or if the individual was previously or currently on treatment.

### Cause of death data

Cause of death data were derived from verbal autopsy interviews. Verbal autopsy (VA) is an indirect method of ascertaining cause of death from information on symptoms, signs and circumstances preceding the death, obtained from primary caregivers and close relatives of the deceased [33]. Each case of death that occurs in the NUHDSS is tracked down, identified and recorded via VA. A special software, the InterVA, uses a probabilistic approach to generate cause of death for verbal autopsy data. The software has been used in different settings in Africa and Asia, details of whose development, validation and use for determining cause of deaths are described elsewhere [34,35,36]. Notably, InterVA-4, the version used in this analysis, follows the cause of death codes defined in the WHO 2012 verbal autopsy instrument which correspond to ICD-10 categories [33]. In this case, the specific ICD-10 codes for acute cardiac disease (I20–I25), stroke (I60–I69), and other unspecified cardiac diseases (I00–I09; I10–I15; I26–I52; I70–I99) including rheumatic diseases were used.

### Exclusion criteria

The CVD 2008 study, NUHDSS residency data, cause of death data datasets were merged. Individuals aged below 30 years (1,164) were excluded from the current analysis given low risk for CVD mortality among such individuals, i.e. no CVD death was recorded from individuals aged 18–30 over the 10-year study period.

## Data analysis

### Imputation of missing height and weight data

Multiple imputation of missing data was carried out on 106 out of 4290 records (about 2.5%) with missing height and weight values. Multivariate normal imputation (MVNI) method in Stata (Stata Corporation, College Station, Texas) was used to create 10 imputations per missing value. The assumption was that the variables with missing data followed a multivariate normal distribution and the missing data were completely random i.e. missing-ness was not related to the outcome of interest. Age, sex and marital status were key anchor variables used in the imputation. After imputation, we compared the means of height and weight variables in the datasets with and without imputation and confirmed that they were similar (see supplementary Figure 1).

### Descriptive and advanced analysis

We describe the distribution of sociodemographic and CVD risk factors in the population, stratified by whether individuals died from CVD or not. Using survival analysis, a multivariable regression approach, we evaluated the association of important prognostic baseline factors with CVD mortality within a 10-year period following baseline risk assessment. Using Cox proportional-hazards regression, we estimated the relative risk of dying from a CVD outcome from physiological and behavioral risk factors, and socioeconomic factors measured at baseline (2008). Model 1, adjusted for age and sex, included physiological risk factors diabetes mellitus, hypertension, total blood cholesterol, overweight and obesity, together with behavioral risk factors current smoking, and physical activity. Education and employment, the two individual-level variables for socioeconomic status were added in model two. At every step, variables that were not statistically significant at p-value < 0.20 were excluded. A cut-off of p = 0.20 for inclusion into the next stage in multivariable modelling was selected based on a common practice for such analysis, where at this significance level the variable has a ‘reasonable association with the outcome’ [37]. Hazard ratios (HR) together with their 95% confidence intervals (95% CI) are reported.

Attributable risk in a population is a function of the prevalence of the risk factor and the strength of its association (relative risk) with the outcome (in this case CVD mortality).

{\rm{PAR}} = {\rm{P}}_{\rm{e}}({\rm{RR}}_{\rm{e}}- 1)/[1 + {\rm{P}}_{\rm{e}}({\rm{RR}}_{\rm{e}}- 1)],

where P_e_ is the prevalence of the risk factor, and RR_e_ is the relative risk of mortality due to that risk factor.

We estimated post-Cox regression the reduction in incidence of the event (death from CVD over the 10-year period) that would result if the prognostic baseline risk factor was completely eliminated from the population, relative to a situation when the risk factor was present, or simply put, the population attributable fraction (PAF). We included factors that were significantly associated with a death from CVD in the Cox regression model. The adjusted PAF represents the reduction in incident CVD deaths if the risk factor was eliminated from the population while adjusting for the effect of the other risk factors or confounders. Data were analyzed using Stata 15.1 (Stata Corporation LP, College Station, TX).

### Ethical considerations

The Scientific and Ethics Review Unit of the Kenya Medical Research Institute (KEMRI) approved the CVD study (SERU/NON-SSC 339). Individual informed consent was sought from all study participants. Participants were made aware that their participation was voluntary, and that they had the freedom to withdraw consent at any point during their participation in the study. The NUHDSS is approved by the Government of Kenya and ethical responsibilities are overseen by KEMRI.

## Results

### Characteristics of study participants

The mean age of the 4290 individuals (44% female) was 48.4 years in 2008. More than half (55.8%) possessed primary school level education and higher. The prevalence of diabetes and hypertension was 7.8% and 24.9% respectively. Of the 9.7% individuals who were obese based on body mass index (BMI) cut-off of >30 Kg/m^2^, the majority (84.5%) was female. Of the 12.2% who were currently smoking, nearly all (98%) were men.

Of the 385 deaths recorded during the 10-year follow-up period (2008–2018), 101 (26%) resulted from CVD. Individuals who died from CVD were older (mean age 64.0 vs 48.1 years), and mostly women (54.5%). Twice as many individuals with diabetes (15.9% vs 7.6%), twice as many individuals with hypertension (54.5% vs 24.2%) died from CVD. Fewer individuals with higher levels of education (19.8% vs 56.7%) died from CVD. A lot more individuals in informal employment died from CVD (65.4%) when compared to unemployed (31.7%) and the formally employed (3.0%). The proportion who died from CVD who were physically inactive was 37.6% compared to 17.3% among those who did not die from CVD. Additional differences between individuals that died from CVD and those who did not die based on baseline characteristics are summarized in Table 1.

**Table 1:** Baseline characteristics of study participants.

Variables	CVD death = 0	CVD death = 1

	N = 4189	N = 101

	n (%)	n (%)

Age ± SD	48.1 ± 12.2	64.0 ± 14.9
Female	1832 (43.7)	55 (54.5)
Ideal blood cholesterol	3246 (77.5)	72 (71.3)
Normal weight (18.5 – 24.9 Kg/m^2^)	2547 (60.8)	59 (58.4)
Underweight (<18.5 kg/m^2^)	313 (7.5)	12 (11.9)
Overweight (BMI 25–29.9 Kg/m^2^)	931 (22.2)	19 (18.8)
Obese (BMI ≥30 Kg/m^2^)	398 (9.5)	11 (10.9)
Diabetes mellitus	317 (7.6)	16 (15.9)
Hypertension	1013 (24.2)	55 (54.5)
Currently smoking	515 (12.3)	9 (8.9)
Insufficient physical activity	724 (17.3)	38 (37.6)
Primary school education and higher	2375 (56.7)	20 (19.8)
Unemployed	441 (10.5)	32 (31.7)
Informal employment	3122 (74.5)	66 (65.4)
Formal employment	626 (14.9)	3 (3.0)

**Key: CVD death = 0:** *did not die from a CVD/alive*; **CVD death = 1:** *died from a CVD*; **diabetes mellitus:** *previous diagnosis by health care professional and currently or previously on medication, random plasma glucose ≥11.1 mmol/L or fasting plasma glucose 7.0 mmol/L*; **hypertension:** *previous diagnosis by healthcare professional and currently or previously on medication, systolic blood pressure (SBP) ≥ 140 mmHg and/or diastolic blood pressure (DBP) ≥ 90mmHg*; **insufficient physical activity:** *based on self-report of less than 150 minutes/week moderate and 75 minutes/week vigorous intensive activities*.

### Determinants for mortality from cardiovascular diseases

Table 2 presents the analysis for associations between baseline factors with mortality from CVD within 10 years of baseline assessment of the prognostic factors, while Table 3 presents results for factors that were found to be statistically significant in their association with CVD mortality in Nairobi’s slums, in addition to sex, which although not statistically significant, is a key demographic factor together with age of the individual. In the final model, a one-unit increase in the age of the individual increased their relative risk of dying from CVD by eleven percentage points (HR 1.11; 95% CI 1.03–1.20, p = 0.005). Being hypertensive more than doubled the risk of death from CVD (HR 2.19, 95% CI 1.44–3.33, p <0.001). Overweight (HR 0.59, 95% CI 0.35–1.00, p = 0.051) when compared to normal weight was marginally associated with decreased risk of CVD death, as was the case with possessing primary school-level education (HR 0.57, 95% CI 0.33–0.99, p = 0.044) and being formally employed (HR 0.22, 95% CI 0.06–0.75, p = 0.015).

**Table 2:** Determinants of mortality from cardiovascular diseases within 10 years of assessment of risk.

Factor	Model 1		Model 2	

	HR (95% CI)	p-value	HR (95% CI)	p-value

Age	1.11 (1.04–1.20)	0.003	1.11 (1.03–1.19)	0.006
Male	0.77 (0.49–1.20)	0.245	0.97 (0.62–1.51)	0.878
Diabetes	1.35 (0.77–2.35)	0.290		
Hypertension	2.09 (1.37–3.20)	0.001	2.11 (1.43–3.32)	<0.001
Underweight	1.20 (0.63–2.27)	0.580		
Overweight	0.56 (0.33–0.96)	0.034	0.59 (0.34–1.00)	0.049
Obesity	0.70 (0.35–1.39)	0.307		
High cholesterol	1.17 (0.75–1.82)	0.484		
Current smoking	0.99 (0.48–2.05)	0.986		
Insufficient physical activity	1.36 (0.87–2.13)	0.178	1.20 (0.75–1.90)	0.452
Primary school education and higher			0.57 (0.33–0.99)	0.045
Informal employment			0.63 (0.38–1.04)	0.072
Formal/salaried employment			0.23 (0.07–0.80)	0.021

**Table 3:** Sex and baseline factors significantly associated with CVD mortality in Nairobi’s slums.

Factor	HR (95% CI)	p-value

Age	1.11 (1.03–1.20)	0.005
Male	0.94 (0.61–1.45)	0.777
Hypertension	2.19 (1.44–3.33)	<0.001
Overweight	0.59 (0.35–1.00)	0.051
Primary school education and higher	0.57 (0.33–0.99)	0.044
Informal employment	0.60 (0.37–0.97)	0.039
Formal/salaried employment	0.22 (0.06–0.75)	0.015

### Population attributable fraction

A 29% reduction in the incident CVD deaths (PAR 95% CI 11%–42%, p = 0.002) would be achieved if hypertension was fully controlled in this community. Adjusting for the effect of other risk CVD factors included in the final regression model, 27% (PAR 95% CI 9%–42%, p= 0.004) of deaths from CVD in this community would be eliminated. Further, if members of this population attained at least primary school level education, a 39% reduction in CVD deaths (95% CI 5%–60%, p = 0.026) would be achieved after adjusting for the effect of the other factors included in the final regression model. Eradicating unemployment would decrease CVD deaths by 17% (PAR 95% CI 5%–27%, p = 0.035).

## Discussion

In Nairobi’s slums older age and hypertension were associated with increased relative risk of dying from CVD, while favorable socioeconomic indicators, such as being employed and attaining primary school level education and higher were associated with decreased risk of CVD mortality. The link between hypertension and the increased risk for CVD mortality in this community is in line with global trends [38,39].

Smoking and tobacco use, sedentary behavior, insufficient physical activity, harmful alcohol consumption, and insufficient fruit and vegetable intake have been linked to increased risk for CVD morbidity and mortality globally [40,41,42]. However, we did not find an association between tobacco smoking (whose prevalence was skewed to males at 98% among those that smoked tobacco), insufficient physical activity and insufficient fruit and vegetable intake with CVD mortality in this community. It is important to note that members of the urban slum community are generally physically active (only about 17% reported insufficient physical activity), while insufficient fruit and vegetable intake was highly prevalent (up to 74%). The observed lack of association between tobacco smoking and CVD mortality could possibly be attributed to underreporting and unreliability of self-report data, as is usually the case with behaviors that are considered socially undesirable (a concept known as social desirability bias) [43], specifically among female participants [44].

Although previous research has established that excess weight is associated with a significantly increased risk of CVD morbidity and mortality in the general population, the current findings indicate that overweight conferred a protective effect against the risk of death from CVD mortality in this population. The obesity paradox associates overweight and obesity with decreased mortality risk from CVD when compared to normal weight. Another plausible explanation to this observation could be because the urban slum dwellers were generally physically active. Physical activity occurring together obesity and overweight has been known to offset the effect of the latter on CVD mortality [45,46].

Previous research has shown that individual-level financial, occupational, and educational circumstances can attenuate CVD risk [47,48] while low levels of education are associated with misperception of CVD risk, and consequently asserts an influence on the acceptance of, and adherence to treatment for CVD [49,50]. The health belief model of disease risk and mitigation behavior posits that individuals who perceive themselves to be at a higher risk of developing CVD (more likely to be literate) are more likely to make lifestyle adjustments to mitigate risk factors for CVD and consequently reduce their risk of developing CVD, when they encounter appropriate cues for action [50,51].

A socioeconomic gradient has been documented for CVD risk in high income countries [52]. Emerging research from SSA and in Asia also points to this. For instance, the Prospective Urban and Rural Epidemiology (PURE) study has reported widely on the differential distribution of CVD risk factors across levels of SES, education and employment, and in urban and rural populations [53]. Findings from South Africa showed substantial differences between rural and urban populations with regard to CVD, with the urban populations recording the highest prevalence [54]. In the study, however, education of the urban and rural populations did not influence the distribution of the risk factors [54], a finding that is contrary to what was observed in a population of men and women with secondary school education or higher who had lower BMIs, with the women specifically having lower blood pressure and lipid levels and practicing healthier diets when compared to men and women with lower levels of education [55].

Poverty promotes disparities in CVD prevention in LMICs in SSA and Asia. Inequality, a direct consequence of poverty, rural residence and low levels of education impacted the uptake and adherence to drug treatment among patients with coronary heart disease (CHD) and stroke in the two world regions [56,57]. Elsewhere in China, lower levels of education and income were positively associated with an increase in acute myocardial infarction (AMI) risk [58]. Lower levels of education and income were similarly implicated in smoking or tobacco use, low physical activity and the clustering of major CVD risk factors in India [59].

The results of our study are not without some limitations. Data from self-report suffers from unreliability majorly due to underreporting with respect to socially-undesirable practices. The observed no association between tobacco smoking and CVD deaths in this population could have come about as a result of such underreporting. Inaccuracy in estimation is another limitation of self-report data that may affect measures such as of physical activity and sedentary behavior. Using objective measures to corroborate self-reports could solve this problem. The CVD study on risk factors conducted in 2008 was cross-sectional, and some of the behavioral (e.g. physical activity) and physiological (e.g. blood pressure control) risk factors collected during the study may have changed over the 10 years. Additionally, residents of urban slums are a highly mobile population and some of the individuals that out-migrated may have died from CVD outside of the NUHDSS. We could only include in our analysis cardiovascular-related deaths occurring within the slum. A sub-group analysis of baseline characteristics of individuals that out-migrated, including the sociodemographic variables showed that they were not systematically different from those that remained in the study area (supplementary Table 1).

Our analysis combines robust datasets from a baseline survey for CVD risk factors, longitudinal data on residency and migration in the NUHDSS and verbal autopsy data on cause of death. Longitudinal surveillance data supports time-to-event analysis and is appropriate for estimation, with confidence, the strength of association between several baseline factors with mortality outcomes. Availability of such data is rare in SSA. Additionally, cause of death data generated by verbal autopsies (VA) are a useful and the only available method for determining cause of death in settings where vital registration and clinically certified cause-of-death data are missing, are incomplete or inaccurate [60,61], as is the case with the community where the study was conducted.

## Conclusions

The inverse relationship between education and employment with mortality from CVD in this urban poor community highlights the important role socioeconomic factors play in CVD prevention and control, and the need for a holistic approach in urgently addressing them within the broader context of the social determinants of health at the policy, population and individual levels. Enhancing access to education, employment and livelihood opportunities can enhance primordial prevention of CVD, generating high returns on investment for CVD prevention and control. Particularly, targeted interventions to protect individuals in informal employment from occupation-related hazard and risk for developing and dying from cardiovascular diseases are required. Additionally, adequate prevention and management (treatment and effective control) of hypertension through drug and non-drug therapies is a key intervention for CVD morbidity and mortality in this community.

## Data Accessibility Statement

Data can be accessed following a reasonable request to the APHRC through its Microdata portal.

## Additional Files

The additional files for this article can be found as follows:

10.5334/gh.787.s1 Supplementary Figure 1. Comparison of mean estimates for height and weight with 0 (no imputation) and 10 imputations per missing value.

10.5334/gh.787.s2 Supplemental Table 1. Key baseline sociodemographic characteristics of migrants compared with non-migrants.
