# Supplementary material for: Determinants of Mortality from Cardiovascular Disease in the Slums of Nairobi, Kenya
Source: Glob Heart. 2020 Apr 10;15(1):33. doi: 10.5334/gh.787 (PMC7218782; doi:10.5334/gh.787)
Supplement: Supplementary Figure 1. — Comparison of mean estimates for height and weight with 0 (no imputation) and 10 imputations per missing value. [file gh-15-1-787-s1.pdf]

Supplementary Figure 1: Comparison of mean estimates for height and weight with 0 (no imputation) and 10 imputations per missing value

```
. mi xeq 0 10: summarize cvd_weight2 cvd_height2

m=0 data:
-> summarize cvd_weight2 cvd_height2

    Variable |        Obs        Mean    Std. Dev.       Min        Max
-------------+---------------------------------------------------------
 cvd_weight2 |      4,186    63.23421    11.92463         30      165.2
 cvd_height2 |      4,186     163.219    8.494641      101.9        210

m=10 data:
-> summarize cvd_weight2 cvd_height2

    Variable |        Obs        Mean    Std. Dev.       Min        Max
-------------+---------------------------------------------------------
 cvd_weight2 |      4,290    63.23212    11.89678         30      165.2
 cvd_height2 |      4,290     163.212    8.478182      101.9        210
```

Variable	N	Without imputation<br>Mean (SD)	With 10 imputations<br>Mean (SD)
**Weight (Kg)**	4186	63.23 (11.9)	63.23 (11.9)
**Height (cm)**	4290	163.2 (8.5)	163.2 (8.5)
