# Supplementary material for: Determinants of Mortality from Cardiovascular Disease in the Slums of Nairobi, Kenya
Source: Glob Heart. 2020 Apr 10;15(1):33. doi: 10.5334/gh.787 (PMC7218782; doi:10.5334/gh.787)
Supplement: Supplemental Table 1. — Key baseline sociodemographic characteristics of migrants compared with non-migrants. [file gh-15-1-787-s2.pdf]

Supplementary Table 1: Key baseline sociodemographic characteristics of migrants compared with non-migrants

	Migrants (N = 16)		Non-migrants (N = 4290)	
Factor	Female (n=9)	Male (n=7)	Female (n=1887)	Male (n=2403)
Age (SD)	44.2 (9.9)	53.1 (14.9)	47.9 (13.4)	48.9 (11.7)
Married/living together	4 (44.4)	5 (71.4)	832 (44.1)	1424 (59.3)
Primary school and higher	5 (55.6)	4 (57.1)	848 (44.9)	1547 (64.4)
